# Two-Point Left Ventricle Pacing and Cardiac Computed Tomography

**DOI:** 10.1155/2012/347189

**Published:** 2012-09-09

**Authors:** Rafal Mlynarski, Agnieszka Mlynarska, Eugeniusz Pilat, Rafal Gardas, Jacek Wilczek, Maciej Sosnowski

**Affiliations:** ^1^Department of Electrocardiology, Upper-Silesian Medical Centre, ul Ziolowa 45/47, 40-635 Katowice, Poland; ^2^Unit of Noninvasive Cardiovascular Diagnostics, Upper-Silesian Medical Centre, 40-635 Katowice, Poland; ^3^3rd Division of Cardiology, Medical University of Silesia, 40-752 Katowice, Poland

## Abstract

Endocardial leads can potentially cause problems during coronary vessels visualization in multislice computed tomography (MSCT) due to a large number of artifacts. Based on presented case, we conclude that it is possible to perform MSCT of coronary arteries and leads visualization despite coexistence of four endocardial leads.

## 1. Introduction

There are queries about the possibility of coronary angiography in cardiac computed tomography in patients with endocardial and epicardial leads [1]. In fact, metal elements of leads can cause artifacts. There are some report existing, showing that most problems occurred with atrial pacing and right coronary artery (RCA) [2, 3]. Still the challenge are cases with more than two standard leads (right ventricle and right atrium) implanted. Such situation is in cardiac resynchronization therapy (CRT) where additionally left ventricle (LV) lead is implanted [4]. Sometimes the situation is even more complicated.

We present patient with CRT device implanted in 2006 due to heart failure with left bundle branch block (LBBB) and low ejection fraction (EF). During the last followup visit (01.2012), elevated and not stable left ventricle lead threshold (3,25–5,25 V) was confirmed as well as necessity to upgrade system to the implanted cardioverter-defibrillator ICD system—in Holter monitoring presence of nonsustained ventricular tachycardia (nsVT) was confirmed. Both problems we have to resolve during operation. 

Two week later, we extracted RV pacing lead and implanted RV defibrillation lead. We checked parameters of LV lead and confirmed instability of LV threshed. During contrasting of coronary venous tree, lack of lateral veins was confirmed (old LV lead was positioned in posterolateral vein). There was only anterior and anterolateral vein possible to implantation, however, trying to implant LV lead to the anterolateral vein and showing also instable threshold. Finally, second LV lead was positioned in anterior vein with stable electrical parameters. Both leads were connected to the device via Y-type connector.

Computed tomography was performed using an Aquilion 64 scanner (Toshiba Medical Systems, Japan). Scanning with retrospective ECG gating was performed during a breath hold using 64 slices with a collimated slice thickness of 0.5 mm. A breath-hold examination was performed to adjust the scanner settings. The helical pitch was 12.8 in best mode and the rotation time was 0.4 s. The tube voltage was 135 kV at 380 mA. We used a preselected region of interest (ROI) in the descending aorta. Triggering started at 180 Hounsfield units. 90 ml of nonionic contrast agent (Ultravist 370, Schering, Germany) was given at an rate of 4.5 mL/s. The contrast agent was given in three phases: 90 mL of contrast agent (average), then 24 mL of contrast agent followed by 16 mL of saline flush (60%/40%), and finally 30 mL of saline. During scanning, patient had stable biventricular pacing rhythm 65 beats per minute. Reconstructions of data were performed on Vitrea 2 workstations (Vital Images, USA; software version 5.1). 3D volume rendering (VR) reconstructions, and multiplanar reformatted (MPR) reconstructions were created.

**Figure 1:**
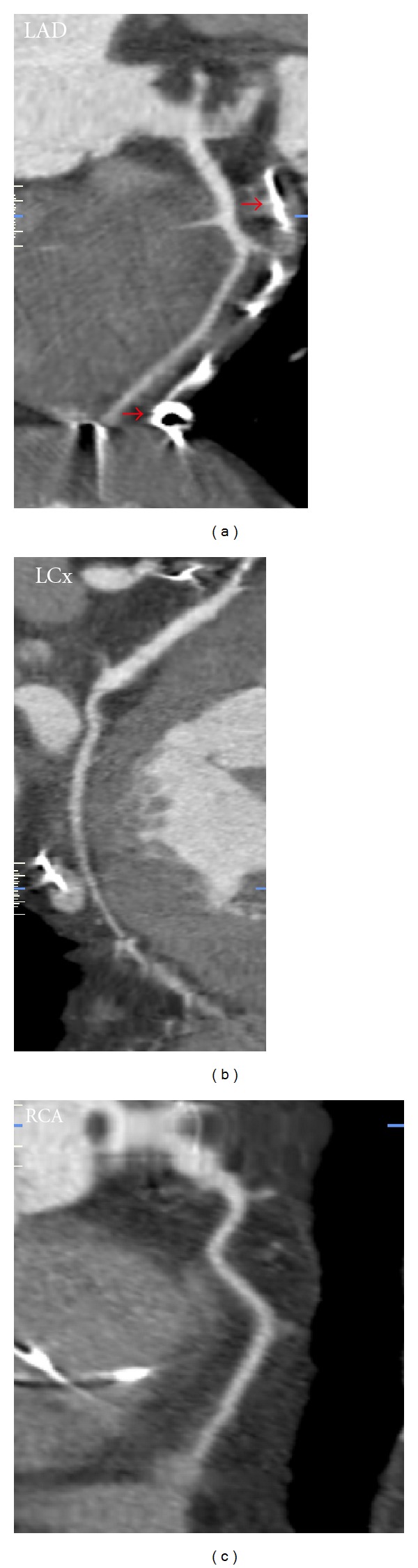
Visualization of coronary arteries (MPR). Red arrows mark distal part of LV lead in anterior vein near LAD.

**Figure 2:**
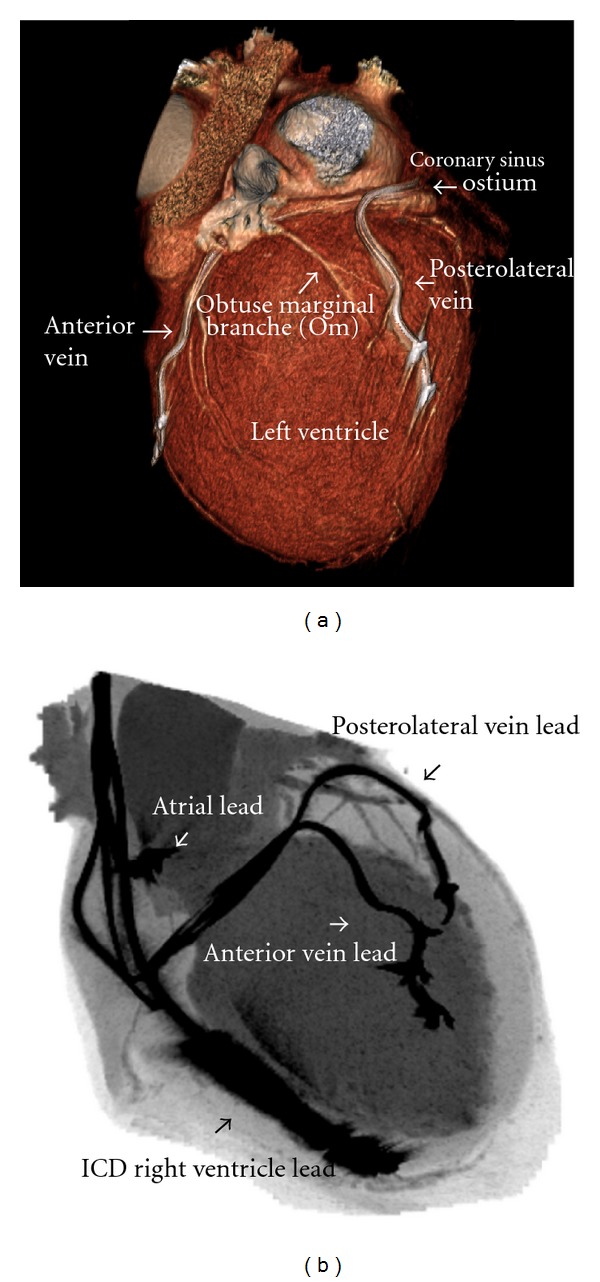
Computed tomography leads visualization: (a) 3D lateral view of the heart (volume rendering). Both LV leads are clearly visible. (b) Own reconstruction similar for intraoperational fluoroscopy. All leads visible.

The result of this examination have not shown the changes in coronaries—Figure 1. Parallel we create visualization of leads without artifacts and interaction with arteries—Figure 2. Important observation is that it was possible to obtain diagnostic images of coronary arteries, despite the presence of multiple endocardial lead.

## 2. Conclusions

 It is possible to perform computed tomography of coronary arteries with parallel visualization of multiple endocardial leads.
